# Influence of childhood socioeconomic position and ability on mid-life cognitive function: evidence from three British birth cohorts

**DOI:** 10.1136/jech-2020-215637

**Published:** 2021-02-25

**Authors:** Eoin McElroy, Marcus Richards, Emla Fitzsimons, Gabriella Conti, George B Ploubidis, Alice Sullivan, Vanessa Moulton

**Affiliations:** 1 Department of Neuroscience, Psychology and Behaviour, University of Leicester, Leicester, UK; 2 Centre for Longitudinal Studies, UCL Institute of Education, University College London, London, UK; 3 MRC Unit for Lifelong Health and Ageing at UCL, University College London, London, UK; 4 Institute of Fiscal Studies, London, UK

**Keywords:** cognition, education, longitudinal studies, psychometrics, social inequalities

## Abstract

**Background:**

Childhood socioeconomic position (SEP) is robustly associated with cognitive function later in life. However, it is unclear whether this reflects a direct relationship, or an indirect association via modifiable factors such as educational attainment and occupation. We sought to clarify these associations using retrospectively harmonised data from three ongoing British birth cohorts.

**Methods:**

We analysed data from the 1946 National Survey of Health and Development (n=2283), the 1958 National Child Development Study (n=9385) and the 1970 British Cohort Study (n=7631). Retrospective harmonisation was used to derive equivalent indicators of cognition, SEP, education and occupation across the three cohorts. Structural equation modelling was used to examine the association between childhood SEP and mid-life cognitive function, via childhood cognitive ability, educational attainment and mid-life occupation.

**Results:**

Across all three cohorts, no direct pathways were observed between childhood SEP and mid-life cognitive function. Rather, this association was indirect via the three temporally ordered mediators. In addition, the direct pathway between childhood cognition and adult cognitive function was weaker in the two younger studies.

**Conclusions:**

Across three British birth cohorts, we found that the association between early life SEP and mid-life cognitive function was fully mediated by childhood cognitive ability, educational attainment and occupational status. Furthermore, the association between early cognitive ability and mid-life cognitive function has decreased in younger generations. Therefore, cognitive function in adulthood may be influenced by modifiable factors and societal change.

## Introduction

Childhood socioeconomic position (SEP) is robustly associated with cognitive function later in life,^1^ however, the mechanisms underlying this association remain poorly understood. It is unclear whether low childhood SEP represents a direct risk for lower levels of cognitive function in adulthood,^2^ or whether this association is indirect via time-varying and potentially modifiable mediating variables.^3 4^ Proposed mediators include educational^5^ and occupational attainment (in particular job complexity),^4 6^ whereby early socioeconomic advantage has a positive impact on both domains, which in turn serve as protective factors for later cognitive function.

The inconsistencies in findings can partially be explained by different samples, mediators and statistical methods used in studies. For example, measures of early cognitive ability are often absent,^7^ and a recent study found that the associations between childhood SEP and adult cognitive function reduced when adjusting for cognition at age 12, which suggests that the strength of association may be exaggerated if earlier cognition is not accounted for.^8^ Moreover, findings may be obscured by a lack of focus on pathways between mediating factors themselves (ie, serial mediation).

This study aims to extend the understanding of the specific pathways between childhood SEP and mid-life cognitive function, adjusting for cognitive ability in childhood. Mid-life remains an under-researched period in the study of cognitive function,^9^ despite the fact that cognition shows different patterns of growth and decline throughout adulthood.^10^ We expand on work in this area by leveraging retrospectively harmonised measures of cognition, childhood and adult SEP, education and early life factors across three British cohorts to investigate: (1) whether childhood SEP is related directly to mid-life cognitive function, or indirectly via childhood ability, education and adult occupation, (2) whether cognitive ability in childhood has a greater direct or indirect (via education and adult occupation) influence on later cognitive function and (3) whether these relations are consistent across cohorts.

We use a structural equation modelling (SEM) framework to estimate latent cognitive factors (‘g’), in childhood (age 10/11) and mid-life (age 46–53). We apply multigroup analysis to test the measurement equivalence of our measures of cognition and SEP across cohorts, and to compare direct and indirect pathways between childhood SEP and cognitive function in mid-life.

## Methods

### Data

Our data were from three ongoing British birth cohort studies:

Medical Research Council National Survey of Health and Development (MRC NSHD): The MRC NSHD is a socially stratified sample (initial N=5362) of men and women born to married parents in England, Scotland and Wales in a single week in March 1946. The sample was selected from an initial maternity survey of 13 687 pregnancies, and consisted of all births to non-manual and agricultural families, and a random one-in-four sample from manual families. To date, the participants have been followed 24 times between ages 2 and 68–69 years.^11^

1958 National Child Development Study (NCDS): The NCDS follows the lives of 17 415 people that were born in England, Scotland or Wales in a single week in March 1958. The NCDS started in 1958 as the Perinatal Mortality Survey and captured 98% of the total births in Great Britain in the target week. The cohort has been followed up 10 times between ages 7 and 55.^12^

1970 British Cohort Study: The BCS70 follows the lives of 17 198 people born in England, Scotland and Wales in a single week in March 1970. Participants have since been followed up nine times between ages 5 and 46.^13^

Missing data were imputed using the R package missForest,^14^ which uses an iterative imputation method based on random forests. This non-parametric approach is particularly effective at imputing mixed-type data.^14^ Imputations were conducted separately by cohort to preserve any differences in mean and covariance structures.^15^ All variables used in our main analyses were included in the imputation models. Given there were no auxiliary variables in our imputation models, cases without complete data on our outcome (mid-life cognitive function) were excluded from subsequent analyses following imputation, in line with the ‘impute and delete’ method.^16^ Our analysed imputed samples were n=2283 in the NSHD, n=9385 in the NCDS and n=7631 in the BCS70. As a sensitivity analyses (available on request), we repeated our analyses without imputing, using the default pairwise deletion approach in Mplus,^17^ and our findings were unchanged.

### Measures

#### Cognitive function in adulthood

Our primary outcome variables were four measures of cognitive function (verbal fluency, immediate and delayed verbal memory and visual processing speed) that were administered across the NSHD (age 53), NCDS (age 50) and BCS70 (age 46–47). The exact same tests were administered using identical methods in both NCDS and BCS70, whereas there were minor differences in the administration and scoring of the immediate and delayed recall tests in the NSHD. To mitigate these differences, we retrospectively harmonised these items by converting them to a common scoring metric (for further details see online supplemental table S1).

10.1136/jech-2020-215637.supp1 Supplementary data

### Cognitive ability in childhood

Comparable tests of cognitive ability were administered across the three cohorts when the children were aged 10 (BCS70) and 11 (NSHD; NCDS). The same test of general cognitive ability, comprising of both verbal and non-verbal subscales, was administered in both the NSHD and NCDS. As this test was not included in BCS70, the most conceptually similar measures were used: the British Ability Scales word similarities and matrices tests. Three tests of verbal skills were available across the cohorts—a vocabulary test (NSHD), reading comprehension test (NCDS) and pictorial language comprehension test (BCS70), as well as comparable measures of mathematical knowledge and arithmetic. As all of the cognitive tests were measured on different scales, simple linear transformations were used to place raw scores on comparable metrics (0–50). Further details of these harmonised measures are available elsewhere.^9 18^

### SEP in childhood

We used previously derived harmonised measures of social class and education derived in each of the three cohorts.^19^ Social class variables (assessed when study children were aged 11 in NSHD and NCDS, and aged 10 in BCS) were based on the father’s occupational status as classified under the 1990 Registrar General’s Social Class system: professional, managerial and technical, skilled non-manual, skilled manual, partly skilled and unskilled. These were treated as ordered categorical variables in our analyses, with higher scores reflecting higher SEP. Paternal and maternal education were included as derived variables that reflected whether cohort mothers and fathers completed any postcompulsory education (0=left school at minimum age; 1=remained after compulsory period). For the NSHD, the school leaving age was 14 for study parents, whereas this had increased to 15 by the time of the NCDS.

### Educational attainment

In both the BCS70 (age 30) and NCDS (age 33), highest education attainment was available, based on the UK National Vocational Qualification (NVQ) system. These ordered categorical variables ranged from 0 (‘no qualifications’) to 5 (‘postgraduate or above’). Given the relatively low proportion of participants in level 5, this level was collapsed with level 4 (‘degree or equivalent’) to create an ordinal variable with five levels. A comparable measure was available at age 26 in NSHD, which we retrospectively harmonised to correspond with NVQ levels (online supplemental table S2).

### Mid-life occupational attainment

We used previously derived harmonised measures of occupational attainment, again based on the 1990 Registrar General’s Social Class system detailed above that were available when cohort members were aged either 42 (NCDS/BCS70) or 43 (NSHD) years.^19^

### Additional covariates

Cohort member sex (male as reference category), birth weight (metric kilograms) and breast feeding (0=never; 1=ever) were included as additional covariates in all models.

### Analysis

We examined the measurement equivalence of latent cognitive function and SEP variables across the cohorts by testing for metric invariance using multigroup confirmatory factor analysis. The available cognitive tests were treated as measured indicators of latent cognition variables (separate variables in childhood and mid-life). Father’s occupation and parental education were used as indicators of a latent childhood SEP variable. First, we estimated a configural model in which all measurement parameters were estimated freely across the three cohorts. Model fit was assessed using the χ^2^ statistic, the comparative fit index (CFI)^20^ and root mean square error of approximation (RMSEA),^21^ with CFI values of greater than 0.90 and RMSEA values of less than 0.08 indicating acceptable fit.^22^ A metric invariant model was then estimated by holding the factor loadings equal across cohort groups. This was then compared with the configural model, by examining differences in fit statistics. Based on established guidelines, values of ∆RMSEA <0.015 and ∆CFI <0.01 were judged to support metric invariance.^23^

**Figure 1:**
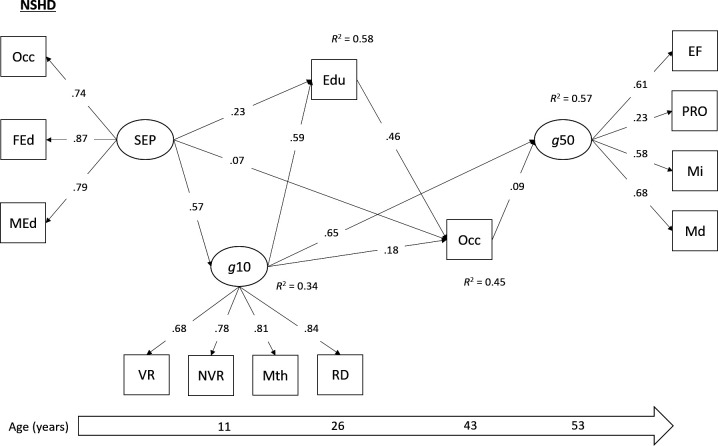
Standardised parameter estimates from multigroup SEM (NSHD group). All paths significant at p<0.05. Model adjusted for sex, birth weight and breast feeding. Edu, harmonised educational attainment; EF, executive functioning; FEd, father’s education; g10, cognitive function in childhood (age 10/11); g50, cognitive ability in mid-life (age 46–53); Md, memory (delayed); MEd, mother’s education; Mi, memory (immediate); Mth, mathematics test; NSHD, National Survey of Health and Development; NVR, non-verbal reasoning; Occ, harmonised measures of occupation (Registrar General’s Social Class); PRO, processing speed; Rd, reading ability; SEP, socioeconomic position; VR, verbal reasoning.

**Figure 2:**
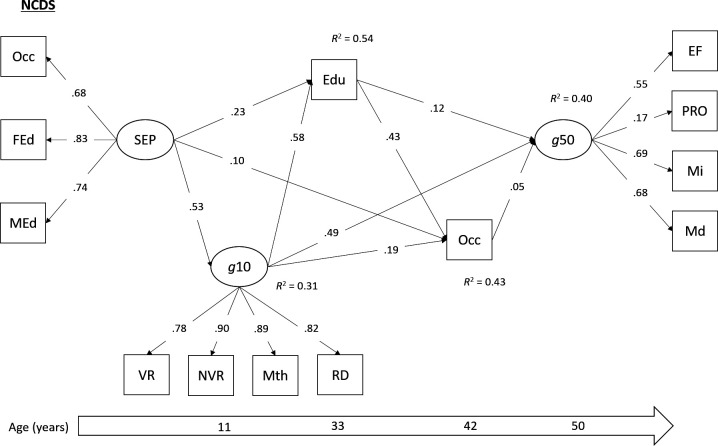
Standardised parameter estimates from multigroup SEM (NCDS group). All paths significant at p<0.05. Model adjusted for sex, birth weight and breast feeding. Edu, harmonised educational attainment; EF, executive functioning; FEd, father’s education; g10, cognitive function in childhood (age 10/11); g50, cognitive ability in mid-life (age 46–53); Md, memory (delayed); MEd, mother’s education; Mi, memory (immediate); Mth, mathematics test; NSHD, National Survey of Health and Development; NVR, non-verbal reasoning; Occ, harmonised measures of occupation (Registrar General’s Social Class); PRO, processing speed; Rd, reading ability; SEP, socioeconomic position; VR, verbal reasoning.

**Figure 3:**
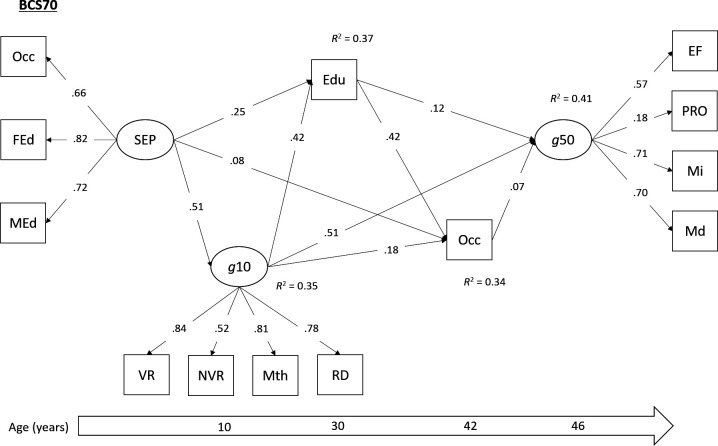
Standardised parameter estimates from multigroup SEM (BCS70 group). All paths significant at p<0.05. Model adjusted for sex, birth weight and breast feeding. BCS, British Cohort Study. BCS, British Cohort Study, Edu, harmonised educational attainment; EF, executive functioning; FEd, father’s education; g10, cognitive function in childhood (age 10/11); g50, cognitive ability in mid-life (age 46–53); Md, memory (delayed); MEd, mother’s education; Mi, memory (immediate); Mth, mathematics test; NVR, non-verbal reasoning; Occ, harmonised measures of occupation (Registrar General’s Social Class); PRO, processing speed; Rd, reading ability; SEP, socioeconomic position; VR, verbal reasoning.

Direct and indirect pathways between childhood SEP and mid-life cognitive function were tested using multigroup SEM. SEM is used to examine the relationships between latent variables, or between latent and observed variables, by combining factor analysis and regression analysis.^24^ SEM can be used to estimate direct and indirect (ie, mediating) pathways simultaneously.^24^ Our tested model (figures 1–3) was both theoretically informed and dictated by the temporal ordering of our study variables. The main exposure variable was SEP in early childhood, a latent variable using the three harmonised indicators (father’s occupation, father’s education, mother’s education). Our primary outcome variable was the latent cognitive function (‘g*’*) variable in mid-life. The three mediating variables included in this serial mediation model were: the latent childhood ability variable, harmonised educational attainment and the harmonised adult occupational measure. Cohort member sex, birth weight and breast feeding were included as observed covariates. As metric invariance was supported in our measurement model, factor loadings were held equal across the three cohorts in our multigroup SEM, to ensure the same latent constructs were being measured and that regression coefficients could be meaningfully compared. Bootstrapped 95% CIs were calculated for direct and indirect effects using 1000 bootstrapped draws, and paths were compared across the cohorts using the Wald test. All models were estimated in Mplus V.8.3,^17^ using the robust weighted least squares estimator due to the categorical nature of several of the variables. Analyses in the NSHD were conducted using the available sampling weight to account for the socially stratified design.

## Results

### Summary statistics

Sample characteristics and descriptive statistics for each cohort are presented in table 1. The distribution of the fathers’ occupation varied across the cohorts. In terms of parental education, the BCS70 had the highest proportion of mothers and fathers who remained in education after the compulsory period. The two younger cohorts had notable increases in educational attainment (as measured by NVQ levels) compared with the NSHD. The breakdown of mid-life occupation of study members also varied by cohort.

**Table 1:** Sample characteristics and descriptive statistics by cohort

	NSHD			NCDS			BCS		
	Mean/count	SD/%	95% CI	Mean/count	SD/%	95% CI	Mean/count	SD/%	95% CI
Covariates									
Sex									
Male	1151	50.42	48.3 to 52.4	4614	49.16	48.1 to 50.2	3646	47.78	46.7 to 48.9
Female	1132	49.58	47.5 to 51.7	4771	50.84	49.8 to 51.9	3985	52.22	51.1 to 53.3
Birth weight (grams)	3397.3	505.56	3376.5 to 3418	3335.69	499.81	3325.6 to 3345.8	3315.29	517.17	3303.7 to 3326.9
Breastfed									
Never	488	21.38	19.7 to 23.1	2608	27.79	26.9 to 28.7	4706	61.67	60.6 to 62.8
Ever	1795	78.62	76.9 to 80.3	6777	72.21	71.3 to 73.1	2925	38.33	37.2 to 39.4
Early life SEP									
SEP (childhood)									
I	160	7.01	6 to 8.1	432	4.6	4.2 to 5	397	5.2	4.7 to 5.7
II	462	20.24	18.6 to 21.9	2053	21.9	21 to 22.7	2058	27	26 to 29
III.1	336	14.72	13.3 to 16.2	927	9.9	9.3 to 10.4	749	9.8	9.2 to 10.5
III.2	783	34.3	32.3 to 36.3	4101	43.7	42.7 to 44.7	3196	41.9	40.8 to 43
IV	430	18.83	17.2 to 20.5	1198	12.8	12.1 to 13.4	862	11.3	10.6 to 12
V	112	4.91	4.1 to 5.9	674	7.2	6.7 to 7.7	369	4.8	4.4 to 5.3
Paternal education									
Compulsory	1534	67.2	65.2 to 69.1	6851	73	72.1 to 73.9	4686	61.4	60.7 to 62.9
Postcompulsory	749	32.8	30.9 to 34.8	2534	27	26.1 to 27.9	2945	38.6	37.4 to 39.7
Maternal education									
Compulsory	1608	70.4	68.5 to 72.3	6824	72.7	71.8 to 73.6	4718	61.8	60.7 to 62.9
Postcompulsory	675	29.6	27.7 to 31.4	2561	27.3	26.4 to 28.2	2913	38.2	37.1 to 39.3
Cognitive ability age 10/11									
Verbal reasoning	30.67	10.61	30.2 to 31.1	29.46	10.78	29.2 to 29.7	29.57	5.57	29.4 to 29.7
Non-verbal reasoning	28.15	8.69	27.8 to 28.5	27.61	8.65	27.4 to 27.8	29.04	8.59	28.8 to 29.2
Mathematics	27.49	10.89	27 to 27.9	22.65	12.24	22.4 to 22.9	31.91	7.62	31.7 to 32.1
Reading	30.64	6.84	30.4 to 30.9	23.98	8.23	23.8 to 24.1	31.35	4.86	31.2 to 41.5
Education									
None	814	35.7	33.7 to 37.7	1124	12	11.3 to 12.7	817	10.7	10 to 11.4
NVQ level 1	163	7.1	6.1 to 8.3	1376	14.7	14 to 15.4	592	7.8	14 to 15.4
NVQ level 2	476	20.8	19.2 to 22.6	2761	29.4	28.5 to 30.4	2412	31.6	30.6 to 32.7
NVQ level 3	604	26.5	24.7 to 28.3	1392	14.8	14.1 to 15.6	986	12.9	12.2 to 13.7
NVQ level 4/5	226	9.9	8.7 to 11.2	2732	29.1	28.2 to 30	2824	37	35.9 to 38.1
Mid-life occupation									
I	136	6	5 to 7	461	4.9	4.5 to 5.4	409	5.4	4.9 to 5.9
II	855	37.5	35.5 to 39.5	3382	36	35.1 to 37	3270	42.9	41.7 to 44
III.1	544	23.8	22.1 to 25.6	2135	22.7	21.9 to 23.6	1495	19.6	18.7 to 20.5
III.2	412	18	16.4 to 19.7	1873	20	19.2 to 20.8	1320	17.3	16.4 to 18.2
IV	255	11.2	9.9 to 12.5	1246	13.3	12.6 to 14	1019	13.4	12.6 to 14.1
V	81	3.5	2.8 to 4.4	288	3.1	2.7 to 3.4	118	1.5	1.3 to 1.8
Mid-life cognitive ability									
Animal naming	23.82	6.63	23.6 to 24.1	22.30	6.26	22.2 to 22.4	23.65	6.14	23.5 to 23.8
Processing speed	280.19	75.53	277.1 to 283.3	333.97	88.81	332.2 to 335.8	346.29	84.38	344.4 to 348.2
Immediate recall	5.79	1.99	5.7 to 5.9	6.55	1.47	6.5 to 6.6	6.64	1.43	6.6 to 6.7
Delayed recall	8.00	2.05	7.9 to 8.1	5.42	1.83	5.4 to 5.5	5.49	1.8	5.4 to 5.5

Data are from imputed sample (n=2283 in NSHD, n=9385 in NCDS and n=7631 in BCS70). Summary statistics for unimputed samples available in online supplemental materials table S3.

BCS, British Cohort Study; NCDS, National Child Development Study; NSHD, National Survey of Health and Development; NVQ, National Vocational Qualification; SEP, socioeconomic position.

### Direct and indirect pathways between SEP and mid-life cognitive function

The multigroup SEM demonstrated acceptable levels of model fit (χ^2^=6479.08, df=268, p≤0.01; RMSEA=0.060; CFI=0.927). The models with and without factor loadings held equal showed little difference in fit (∆RMSEA <0.015, ∆CFI <0.01), which suggested that the measurement parameters were equivalent across the cohorts. As such, metric invariance was supported, and meaningful comparisons of covariances could be made across the three studies. Standardised parameter estimates from this model (with factor loadings held equal to ensure metric invariance) are presented separately for each cohort in figures 1–3, and total and indirect associations are presented in table 2.

**Table 2:** Total, direct and indirect effects from childhood SEP to mid-life cognitive function

Effect	Cohort										β	SE	95 % CI
Total	NSHD										0.470	0.027	0.411 to 0.528
	NCDS										0.341	0.013	0.313 to 0.371
	BCS										0.341	0.015	0.311 to 0.374
Direct	NSHD										0.056	0.038	−0.033 to 0.126
	NCDS										−0.005	0.017	−0.047 to 0.028
	BCS										−0.008	0.021	−0.050 to 0.034
Total indirect	NSHD										0.413	0.028	0.364 to 0.487
	NCDS										0.346	0.010	0.324 to 0.369
	BCS										0.349	0.012	0.323 to 0.376
Specific indirect	NSHD	SEP	→	g10	→	Education	→	Occupation	→	g50	0.014	0.006	0.001 to 0.026
	NCDS										0.006	0.002	0.002 to 0.010
	BCS										0.006	0.001	0.003 to 0.009
	NSHD	SEP	→	g10	→	Occupation	→	g50			0.009	0.004	0.002 to 0.020
	NCDS										0.005	0.001	0.002 to 0.008
	BCS										0.006	0.001	0.003 to 0.090
	NSHD	SEP	→	Education	→	Occupation	→	g50			0.010	0.004	0.001 to 0.018
	NCDS										0.005	0.001	0.001 to 0.008
	BCS										0.006	0.002	0.003 to 0.010
	NSHD	SEP	→	g10	→	Education	→	g50			0.001	0.018	−0.038 to 0.036
	NCDS										0.038	0.006	0.027 to 0.050
	BCS										0.025	0.004	0.017 to 0.035
	NSHD	SEP	→	g10	→	g50					0.374	0.032	0.318 to 0.461
	NCDS										0.257	0.010	0.239 to 0.281
	BCS										0.273	0.012	0.249 to 0.301
	NSHD	SEP	→	Education	→	g50					0.001	0.013	−0.025 to 0.029
	NCDS										0.029	0.005	0.021 to 0.040
	BCS										0.028	0.005	0.018 to 0.038
	NSHD	SEP	→	Occupation	→	g50					0.006	0.004	−0.001 to 0.017
	NCDS										0.005	0.002	0.002 to 0.008
	BCS										0.005	0.002	0.002 to 0.009

BCS, British Cohort Study; NCDS, National Child Development Study; NSHD, National Survey of Health and Development; SEP, socioeconomic position.

A similar pattern of direct and indirect associations emerged. In all three cohorts, the association between childhood SEP and mid-life cognitive function was fully mediated by childhood cognitive ability, educational attainment and mid-life occupation. Indeed, a significant serial mediation effect was found from childhood SEP to adult cognition via all three mediators, and this association did not differ across cohorts (Wald χ^2^=1.39, p=0.49). Additional serial mediation associations were observed via different combinations of these mediating variables (table 2). Three notable differences were observed between the NSHD and the two younger cohorts. First there was no significant direct path between adult educational attainment and subsequent cognitive functioning in the NSHD. However, educational attainment did sit on the serial mediation path between childhood SEP and adult cognitive function via adult occupational attainment in this cohort. Furthermore, when testing this direct pathway using multigroup confirmatory factor analysis (CFA), the Wald test indicated no significant difference across cohorts (Wald χ^2^=4.78, p=0.09). Second, the direct effect of childhood cognitive ability on later cognitive function, which was consistently the strongest direct effect across all cohorts, was significantly stronger in the NSHD compared with the other cohorts (Wald χ^2^=160.13, p<0.01). This may account for the larger proportion of variance that is explained in midlife cognition in the NSHD. Third, the path from cognitive ability in childhood to later educational attainment was weaker in BCS70 (Wald χ^2^=17.36, p<0.001).

## Discussion

The present study used harmonised measures across three British birth cohorts to investigate specific pathways from childhood SEP to mid-life cognitive function, via mediators established in the existing literature (childhood cognitive ability, adult educational and occupational attainment). Specifically we sought to test (1) whether childhood SEP was directly associated with mid-life cognitive function, or whether this antecedent operates indirectly through childhood cognition, and subsequent education and adult occupation, (2) whether cognitive ability had a greater direct influence on adult cognitive function, or indirect via education and adult occupation and (3) whether these relations were similar across cohorts.

After having established measurement equivalence across the studies, an extremely consistent pattern emerged in all three cohorts in which childhood SEP was not directly associated with mid-life cognitive function. Rather, childhood SEP was indirectly associated with later cognitive outcomes via all three temporally ordered mediators.^3 4^ These results go some way to reconciling previously inconsistent findings as to whether childhood SEP exerts a direct influence on mid-life cognitive function.^2 6 8^ The mixed findings of previous studies could be attributable, at least in part, to the exclusion of early cognitive ability,^6^ and different cultural contexts.^2 8^ In this study, however, we consistently found no direct influence of SEP on mid-life cognitive function across three different generations in Great Britain.

These indirect paths demonstrate a long-lasting influence by which childhood SEP was associated with later educational and occupational outcomes, which in turn predicted cognitive function in mid-life. Importantly, across all three birth cohorts, indirect associations were found via education and occupational attainment while controlling for early life cognitive ability. This suggests that cognitive function in mid-life can be impacted by modifiable factors across the life course, and is not purely the continuation of childhood cognition. Indeed, both education and adult occupation are thought to build ‘cognitive reserve’, the brain’s ability to make flexible and efficient use of cognitive networks to enable a person to continue to carry out cognitive tasks despite brain changes.^25^ The number of years of formal education^26^ and having a mentally stimulating or complex job^27^ may help to build cognitive reserve. The influence of education should also be viewed in relation to its impact on determining adult occupation, which in turn influences cognitive function in mid-life.

Looking at the direct pathways, early life cognition was consistently the strongest predictor of mid-life cognitive function. This is in line with the evidence for the stability of individual differences in cognitive function throughout most of the lifespan,^28^ thus, highlighting the importance of cognitive function with respect to one’s cognitive reserve level, even prior to early adulthood. However, the direct pathway was weaker in the NCDS and BCS70 compared with the older NSHD. Furthermore, a direct effect between education and later cognitive function was not observed in the oldest cohort (although direct cross-cohort comparisons indicated no significant difference between these paths). These cross-cohort differences tentatively suggest a changing influence over time, in which education has become an increasingly influential determinant of later cognitive function, and childhood ability less so. These differences might be explained by the major changes across the second half of the 20th century in the economic and social structure of society and major shifts in policies and practices in both health and education. During the early postwar years, as in the NSHD, parental education was lower, nutrition was controlled by food rationing, and opportunities for higher education and subsequently occupation were limited.^29^ The stronger pathway from cognition in childhood to adulthood in the NSHD, which accounted for considerable variance in adult cognition, could reflect improvements in health and increased educational opportunities in earlier life for the more recent cohorts.^30^

Consistent with other studies using the British cohorts, we found that, the influence of childhood cognitive ability on education declined over time.^31^ This finding supports previous claims that those who benefited the most from the aforementioned educational reforms were less able children.^32^ The Education Act of 1972, increased the school leaving age from 15 to 16 in the UK, resulting in an expansion of educational opportunities for the younger cohorts.^30^ In addition, by the 1980s, most British students were being taught in mixed ability schools. The old selective system may have placed greater emphasis on cognitive ability, which directly determined their educational opportunities. However, no change was observed on the direct influence of childhood SEP on educational attainment or adult occupation.^31^ Although the absolute rates of upward mobility have changed in the middle decades of the 20th century as a result of the sizeable expansion of ‘white collar’ and resulting reduction of ‘blue collar’ jobs,^33^ the empirical research on whether social mobility has increased, decreased or stayed the same in the latter decades of the 20th century is inconsistent.^34 35^

Also, it must be noted that our measures of cognitive function in adulthood are considered to reflect fluid cognition. Using a measure of crystallised cognition, thought to be more stable across mid-life, may have resulted in greater associations with education across all three cohorts. Indeed, Richards and Sacker^3^ found a stronger relationship using the National Adult Reading Test (NART) in the NSHD, than measures of fluid cognition. Furthermore, in order to maximise equality of measurement, we treated cognition as a latent general construct, whereas other studies have found that education is more strongly related to specific cognitive skills.

The findings discussed above should also be considered in light of both strengths and limitations of the present study. With our observed mediators, although we retrospectively harmonised these variables, it was not possible to empirically test their measurement equivalence. A second limitation is that the cognitive measures, although administered at similar developmental periods, were completed at different ages across cohorts (maximum difference of 1 year in childhood; 6 years difference in adulthood). Although such age differences could bias mean comparisons (not a primary aim of this study), it is unclear the extent to which they could influence covariances. In addition, our samples were solely from Great Britain, therefore, findings may not generalise to societal, educational and labour market contexts in other cultures. Furthermore, there are unobservable confounders, for example, parental cognition due to the heritable component of both general cognitive function and SEP, and genetics which may partially explain the relation between childhood SEP and cognition.^36^ However, Richards *et al*,^37^ using the NSHD found no relation between APOEe4 (the best known genetic risk factor for clinically significant decline)^38^ and cognitive ability at age 8 or in the NART at age 53. In addition, there may be other omitted variables which might be related to both social position and cognition, for example, cardiovascular health and physical activity.^39^

Nevertheless, the study benefits from several strengths. The inclusion of three British birth cohorts mean our results are generalisable across different historical contexts. Furthermore, the use of representative samples of the British population, along with prospectively collected data in early and mid-life are additional strengths. Also the same measures of cognitive function (memory, verbal fluency and processing speed) were captured in mid-life, and these measures were used to construct a latent variable, allowing for comparisons across three cohort generations. Moreover, not only did we include consistent and harmonised measures across these cohorts, but where possible we explicitly tested the measurement equivalence of our variables using multigroup CFA. Indeed, as metric invariance was supported in both childhood and adult measures of cognition, we were able to compare the strengths of associations across cohorts with confidence. Also, our SEM modelling framework allowed us to account for measurement error in our cognitive variables, and early-life SEP.

In conclusion, across three British birth cohorts, we found that the path from early life SEP to mid-life cognitive function was fully mediated by childhood cognitive ability, educational attainment and occupation status. However, cross-cohort differences were observed in the influence of educational attainment; higher attainment was associated with improved cognitive function in mid-life for the more recent cohorts. Reducing the risk of cognitive decline in later life may start at sensitive periods in early childhood which are modifiable, and further influenced by individual life choices and societal changes across the life course.

**What is already known on this subject**

- Socioeconomic position (SEP) in childhood is associated with cognitive function later in life.
- Failing to control for childhood cognitive ability may inflate this association.
- It is unclear whether childhood SEP directly impacts later cognitive function, or whether it operates via modifiable factors (ie, mediators) across development, such as education and occupation.

**What this study adds**

- Using retrospectively harmonised data, this study examined the association between childhood SEP and mid-life cognitive function in three British birth cohorts, and explored the mediating effect of childhood cognitive ability, adult educational attainment and mid-life occupation.
- Across all three cohorts, there was no direct association between childhood SEP and mid-life cognitive function, rather this association was indirect via all three mediators.
- These findings suggest that the relationship between early life SEP and mid-life cognitive function can be influenced by modifiable factors across the life course.

## Data Availability

Data are available in a public, open access repository. The data underlying this article are available in the UK Data Service repository at https://ukdataservice.ac.uk/.
