# Supplementary material for: Influence of childhood socioeconomic position and ability on mid-life cognitive function: evidence from three British birth cohorts
Source: J Epidemiol Community Health. 2021 Feb 25;75(7):643–50. doi: 10.1136/jech-2020-215637 (PMC8223660; doi:10.1136/jech-2020-215637)
Supplement: Supplementary data [file jech-2020-215637supp001.pdf]

Influence of childhood socio-economic position and ability on mid-life cognitive function – evidence from three British birth cohorts

Eoin McElroy[1*], Marcus Richards[2], Emla Fitzsimons[1], Gabriella Conti[1], George B. Ploubidis[1], Alice Sullivan[1,] Vanessa Moulton[1]

[1] Centre for Longitudinal Studies, University College London, London, United Kingdom

[2] MRC Unit for Lifelong Health, University College London, London, United Kingdom

***Correspondence to Dr Eoin McElroy, Lecturer in Psychology, Department of Neuroscience, Psychology and Behaviour, University of Leicester, UK, email: em447@leicester.ac.uk

Supplementary Materials

Methods S1.

Details of harmonised outcome measures (adult cognitive function)

Several comparable tests (verbal fluency, immediate and delayed verbal memory and visual processing speed) were administered across the NSHD, NCDS and BCS when participants were aged between 46 and 53 years old. The verbal fluency (executive function) test was an animal naming test, in which respondents were given one minute to name as many animals as they could think of. For the letter cancellation tests, participants were presented with blocks of letters, and were asked to read through the blocks from left to right, crossing out the 'Ws' and 'Ps' as they read. Search speed was calculated by summing the total number of words scanned, including both target and non-target words. The immediate and delayed memory trials differed slightly across the cohorts. In the NSHD, participants were shown a list of 15 words at a rate of one word every two seconds. They were then asked to write down as many words as they could recall. This trial was done a total of three times, and a total score was calculated as the sum of the words correctly recalled over the three trials. In both the NCDS and BCS, participants were played an audio recording of 10 words and were then given two minutes to orally recount as many as they could recall. Only one trial was administered. In order to make these variables more comparable, we used the first trial only from the NSHD, and collapsed scores on this variable by recoding scores of greater than 10 to exactly 10. This placed the variable on a similar 0-10 metric that was comparable with the variables in the NCDS and BCS. We did this for both the immediate and delayed conditions. For further details see the supplementary materials.

Table S1. Variables used in cross-cohort psychometric analyses in midlife (age 46-53)

Measure	Cohort	Variable	Harmonisation	N	Mean (SD)	Range
Word List Recall Test	NSHD	wlt199 (immediate memory – 1st trial)	Scores > 10 recoded to a value of 10	2,909	5.80 (2.01)	0-10
		wlt499 (delayed memory – 1st trial)	Scores > 10 recoded to a value of 10	2,292	7.99 (2.05)	0-10
	NCDS	N8CFLISN (immediate)	-	9,648	6.54 (1.48)	0-10
		N8CFLISD (delayed)	-	9,591	5.41 (1.84)	0-10
	BCS	B10CFLISN (immediate)	-	8,501	6.61 (1.44)	0-10
		B10CFLISD (delayed)	-	8,494	5.47 (1.81)	0-10
Animal naming	NSHD	anin	-	2,949	23.56 (6.91)	1-62
	NCDS	N8CFANI	-	9,648	22.28 (6.30)	0-65
	BCS	B10CFANI	-	8,498	23.63 (6.19)	1-70
Letter cancellation	NSHD	CANSP99 (Search speed)	-	2,932	281.07 (76.08)	64-591
	NCDS	N8CFRC (Search speed)	-	9,442	334.10 (88.83)	84-780
	BCS	B10CFRC (Search speed)	-	8,242	346.45 (84.77)	28-780

Table S2. Harmonisation of NSHD education variable to NVQ level.

Qualification	N	Original value	Recoded value
None attempted	1,765	0	0
Vocational course, proficiency only	144	1	1
Sub GCE or sub Burnham C	209	2	1
GCE 'O' level or Burnham C	863	3	2
GCE 'A' Level or Burnham B	610	4	3
Burnham A2	430	5	3
1st Degree or graduate equivalent	369	6	4
Higher degree, Masters	30	7	4
Higher degree, doctorate	12	8	4

Table S3. Sample characteristics and descriptive statistics by cohort (raw data)

			Mean (SD) / Count (%) NSHD	NCDS	BCS70
Covariates	Sex	Male	1151 (50.42)	4614 (49.16)	3646 (47.78)
		Female	1132 (49.58)	4771 (50.84)	3985 (52.22)
	Birthweight (grams)		3397.30 (505.56)	3335.69 (499.81)	3315.29 (517.17)
	Breastfed	Never	488 (21.38)	2608 (27.79)	4706 (61.67)
		Ever	1795 (78.62)	6777 (72.21)	2925 (38.33)
Early life SES	SES (childhood)	I	160 (7.01)	432 (4.6)	397 (5.2)
		II	462 (20.24)	2053 (21.9)	2058 (27.0)
		III.1	336 (14.72)	927 (9.9)	749 (9.8)
		III.2	783 (34.30)	4101 (43.7)	3196 (41.9)
		IV	430 (18.83)	1198 (12.8)	862 (11.3)
		V	112 (4.91)	674 (7.2)	369 (4.8)
	Maternal Education	Compulsory	1534 (67.2)	6851 (73.0)	4686 (61.4)
		Post-compulsory	749 (32.8)	2534 (27.0)	2945 (38.6)
	Paternal Education	Compulsory	1608 (70.4)	6824 (72.7)	4718 (61.8)
		Post-compulsory	675 (29.6)	2561 (27.3)	2913 (38.2)
Cognitive ability age 10/11		Verbal reasoning	30.67 (10.61)	29.46 (10.78)	29.57 (5.57)
		Non-verbal reasoning	28.15 (8.69)	27.61 (8.65)	29.04 (8.59)
		Mathematics	27.49 (10.89)	22.65 (12.24)	31.91 (7.62)
		Reading	30.64 (6.84)	23.98 (8.23)	31.35 (4.86)
Education		None	814 (35.7)	1124 (12.0)	817 (10.7)
		NVQ Level 1	163 (7.1)	1376 (14.7)	592 (7.8)
		NVQ Level 2	476 (20.8)	2761 (29.4)	2412 (31.6)

	NVQ Level 3	604 (26.5)	1392 (14.8)	986 (12.9)
	NVQ Level 4/5	226 (9.9)	2732 (29.1)	2824 (37.0)
Mid-life occupation	I	136 (6.0)	461 (4.9)	409 (5.4)
	II	855 (37.5)	3382 (36.0)	3270 (42.9)
	III.1	544 (23.8)	2135 (22.7)	1495 (19.6)
	III.2	412 (18.0)	1873 (20.0)	1320 (17.3)
	IV	255 (11.2)	1246 (13.3)	1019 (13.4)
	V	81 (3.5)	288 (3.1)	118 (1.5)
Mid-life cognitive ability	Animal naming	23.82 (6.63)	22.3 (6.26)	23.65 (6.14)
	Processing speed	280.19 (75.53)	333.97 (88.81)	346.29 (84.38)
	Immediate recall	5.79 (1.99)	6.55 (1.47)	6.64 (1.43)
	Delayed recall	8.00 (2.05)	5.42 (1.83)	5.49 (1.80)
